# Supplementary material for: Multivariate description of gait changes in a mouse model of peripheral nerve injury and trauma
Source: PLoS One. 2025 Jan 7;20(1):e0312415. doi: 10.1371/journal.pone.0312415 (PMC11706367; doi:10.1371/journal.pone.0312415)
Supplement: S1 File — All confusion matrices are from a single iteration of ten randomly selected training-testing splits. Performance metrics reported in the manuscript are the average of those ten. (DOCX) [file pone.0312415.s001.docx]

**Supporting information**

| **Confusion Matrices of Varying Classifier Architectures**  **(Peripheral Injury vs. Control)** | |
| --- | --- |
| 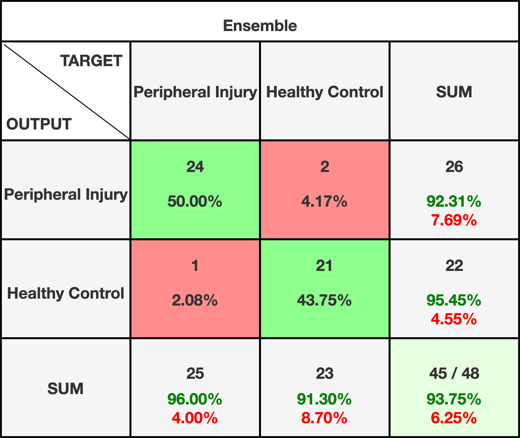 | 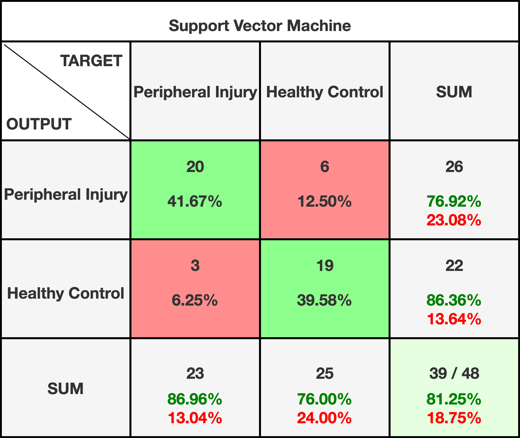 |
| 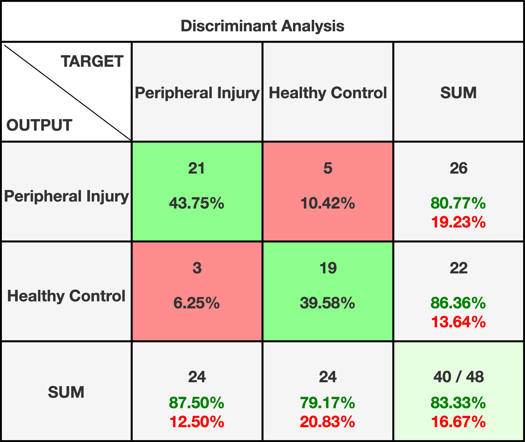 | 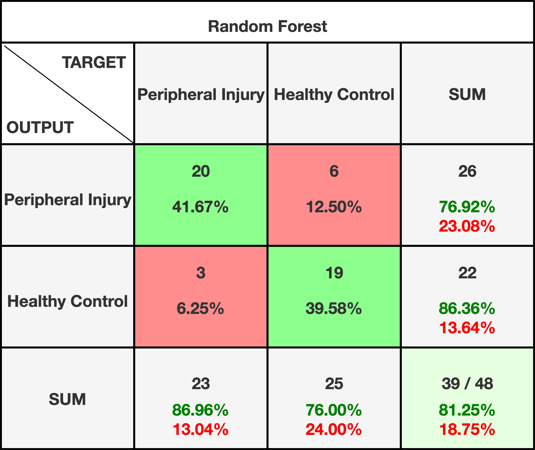 |
| 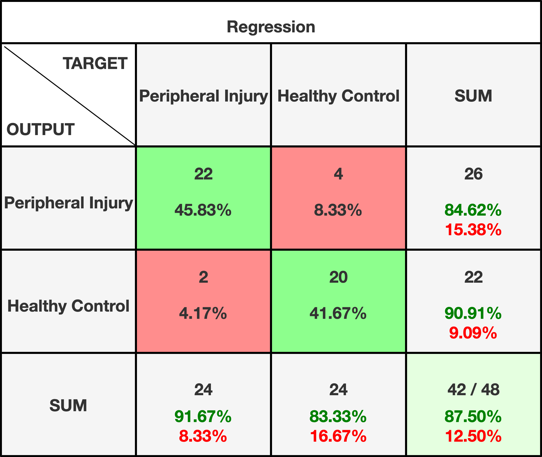 | |

S1 Fig**. Confusion Matrices of Varying Classifier Architectures (Peripheral Injury vs. Control).** All confusion matrices are from a single iteration of ten randomly selected training-testing splits. Performance metrics reported in the manuscript are the average of those ten.

| **Confusion Matrices of Varying Classifier Architectures**  **(Nerve Transection vs. Limb Transplant)** | |
| --- | --- |
| 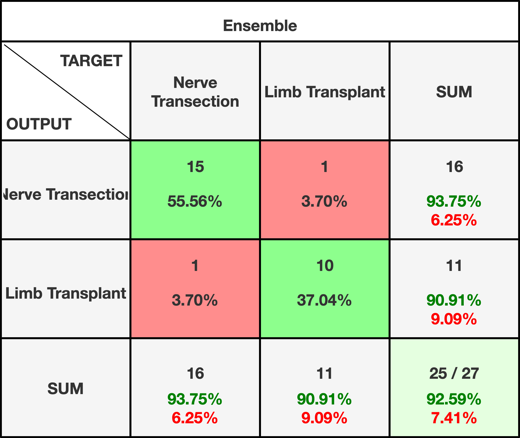 | 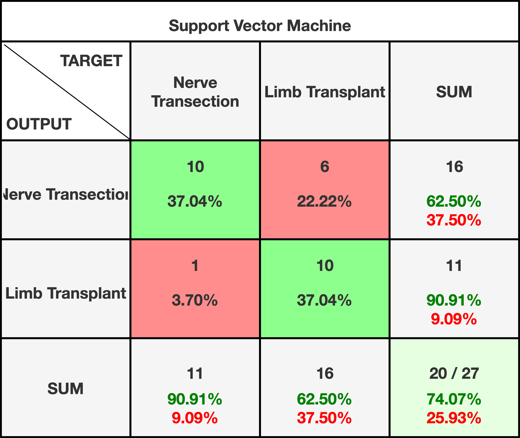 |
| 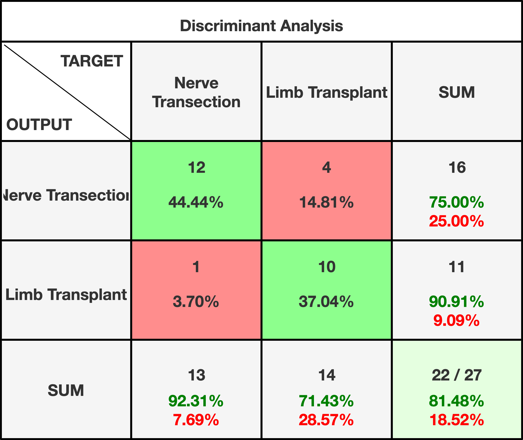 | 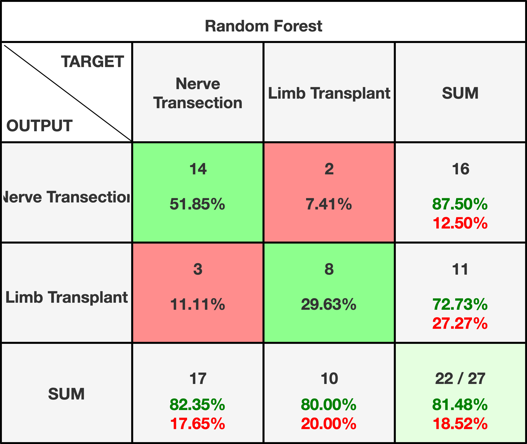 |
| 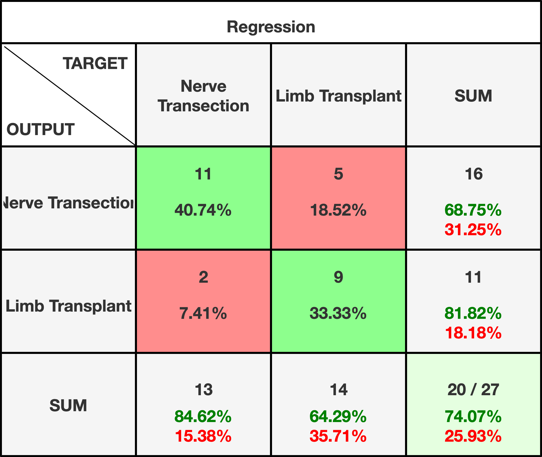 | |

S2 Fig**. Confusion Matrices of Varying Classifier Architectures (Nerve Transection vs. Limb Transplant).** All confusion matrices are from a single iteration of ten randomly selected training-testing splits. Performance metrics reported in the manuscript are the average of those ten.
