# Supplementary material for: Multivariate description of gait changes in a mouse model of peripheral nerve injury and trauma
Source: PLoS One. 2025 Jan 7;20(1):e0312415. doi: 10.1371/journal.pone.0312415 (PMC11706367; doi:10.1371/journal.pone.0312415)
Supplement: Attachment — Submitted filename: Response to Reviewers.docx [file pone.0312415.s002.docx]

Dear Editor,

We are submitting a revised version of our manuscript (# ) entitled “**Multivariate description of gait changes in a mouse model of peripheral nerve injury and trauma**” by Bilal A. Naved, et al. for reconsideration for publication in *PLOS One*.

We thank the Editors and Reviewers for their insightful and constructive comments. To address all concerns and questions raised by the reviewers, we made extensive modifications/clarifications of the manuscript. We believe that our manuscript has considerably improved in quality, and hope that the paper is now acceptable for publication. The following are our point-by-point responses to reviewers’ comments with quoted excerpts included for your convenience:

**Journal Requirements:**

| **Comment from Editor / Reviewer** | **Responses & Quoted Excerpts from Manuscript** |
| --- | --- |
| 1. Please ensure that your manuscript meets PLOS ONE's style requirements, including those for file naming. | Thank you for sharing the style guides. The manuscript has been updated to comply with the shared guides. |
| 2. To comply with PLOS ONE submissions requirements, in your Methods section, please provide additional information regarding the experiments involving animals and ensure you have included details on (1) methods of sacrifice,  and (2) efforts to alleviate suffering | Thank you for reminding us of this requirement. Additional details regarding the total number of animals, number per group, efforts to alleviate suffering, and methods of sacrifice have been added as quoted below:  *“43 male B6 mice (mus musculus), 8 to 10 weeks old (20g), were obtained from The Jackson Laboratory (Bar Harbor, ME, USA) and were group-housed* (lines 487-488)  *… There were three groups: 17 animals in the negative control group received no treatment. 12 animals in the experimental group received only nerve damage (neurorrhaphy) i.e. a complete nerve transection of the femoral and sciatic nerves with re-coaptation. 14 animals in the second experimental group received a total hind-limb transplant* (lines 490-493) *… All surgery was performed with animals kept on a self-regulating heating pad and under isofluorane anesthesia. The depth of anesthesia was examined before surgery with a toe pinch response and subsequent checks at 15-minute intervals during the surgery. Post-operatively, the animals were kept in their cages, with a portion of the cage on a warm pad. The animals were checked on every hour for the first 3 post-op hours and then 4 times a day for two days for any signs of surgical complications. To minimize suffering, pain was managed via local anesthetics (bupivacaine or lidocaine) at the surgical site with a multi-modal analgesic regimen of Buprenorphine-ER and meloxicam to last an additional 3 days. At the conclusion of all experimental timepoints animals were sacrificed via Isofluorane euthanasia with confirmatory bilateral thoracotomy.* (lines 555-564)*”* |
| 3. Thank you for stating in your Funding Statement: "Research reported in this publication was supported by the National Institutes of Health under Award Numbers F30DK123985, and T32GM008152 (BAN); DOD Department of the Army: W81XWH[2110862](tel:2110862) (Zhang), McCormick Foundation/Northwestern Memorial Hospital (Zhang and Werthiem), Julius N. Frankel Foundation via Northwestern Memorial Foundation (Zhang, Han, and Wang); American Heart Association: 20POST35210774 and the Canadian Institute for Health Research: RN409371 - 430628 (Koss); and R01LM013337 (Luo). The content is solely the responsibility of the authors and does not necessarily represent the official views of the National Institutes of Health. Funding sources had no roles in study design, collection, analysis, and interpretation of the data, in the writing of the report, nor in the decision to submit the article for publication.”   Please provide an amended statement that declares *all* the funding or sources of support (whether external or internal to your organization) received during this study, as detailed online in our guide for authors at [http://journals.plos.org/plosone/s/submit-now.](https://urldefense.com/v3/__http://journals.plos.org/plosone/s/submit-now.__;!!Dq0X2DkFhyF93HkjWTBQKhk!XP0w4WqUZQ6ggdbqQcmavHZePLfsYFlXUGFxgbMWuKV1ovzLKi4qCGcXW8MaYWW_xGxL9eCIeGOU18IMJW_5NlA$)  Please also include the statement “There was no additional external funding received for this study.” in your updated Funding Statement. Please include your amended Funding Statement within your cover letter. We will change the online submission form on your behalf. | Understood - all internal and external sources of funding have now been reported in the Funding Statement with the additional confirmatory statement included. The amended Funding Statement has been included in the cover letter and is included below for convenience:  *“Research reported in this publication was supported by the National Institutes of Health under Award Numbers F30DK123985, and T32GM008152 (BAN); CTC Transplant Innovation Endowment grant* [*(110-5442000*](tel:1105442000)*) (Zhang), DOD Department of the Army: W81XWH2110862 (Zhang), McCormick Foundation/Northwestern Memorial Hospital (Zhang and Wertheim), Julius N. Frankel Foundation via Northwestern Memorial Foundation (Zhang, Han, and Wang); American Heart Association: 20POST35210774 and the Canadian Institute for Health Research: RN409371 - 430628 (Koss); and R01LM013337 (Luo). The content is solely the responsibility of the authors and does not necessarily represent the official views of the National Institutes of Health. Funding sources had no roles in study design, collection, analysis, and interpretation of the data, in the writing of the report, nor in the decision to submit the article for publication. There was no additional external funding received for this study.”* |
| 4. When completing the data availability statement of the submission form, you indicated that you will make your data available on acceptance. We strongly recommend all authors decide on a data sharing plan before acceptance, as the process can be lengthy and hold up publication timelines. Please note that, though access restrictions are acceptable now, your entire data will need to be made freely accessible if your manuscript is accepted for publication. This policy applies to all data except where public deposition would breach compliance with the protocol approved by your research ethics board. If you are unable to adhere to our open data policy, please kindly revise your statement to explain your reasoning and we will seek the editor's input on an exemption. Please be assured that, once you have provided your new statement, the assessment of your exemption will not hold up the peer review process. | Thank you for this reminder. We have decided on a data sharing plan and to make use of one of the accepted data repositories (FigShare). Our data has been uploaded to FigShare and the corresponding DOI has been included in Data Availability section as quoted below:  *“The datasets generated during and/or analyzed during the current study are publicly available at a data repository and can be found at the following DOI (10.6084/m9.figshare.25546822).”* (lines 1938-1939) |
| 5. While revising your submission, please upload your figure files to the Preflight Analysis and Conversion Engine (PACE) digital diagnostic tool, [https://pacev2.apexcovantage.com/](https://urldefense.com/v3/__https://pacev2.apexcovantage.com/__;!!Dq0X2DkFhyF93HkjWTBQKhk!XP0w4WqUZQ6ggdbqQcmavHZePLfsYFlXUGFxgbMWuKV1ovzLKi4qCGcXW8MaYWW_xGxL9eCIeGOU18IMSWnxzZ0$). PACE helps ensure that figures meet PLOS requirements. To use PACE, you must first register as a user. Registration is free. Then, login and navigate to the UPLOAD tab, where you will find detailed instructions on how to use the tool. If you encounter any issues or have any questions when using PACE, please email PLOS at [figures@plos.org](mailto:figures@plos.org). Please note that Supporting Information files do not need this step. | Figure files have been uploaded to the PACE tool successfully with no problems identified |

**Reviewer #1**

| **Comment from Editor / Reviewer** | **Responses and Quoted Excerpts from Manuscript** |
| --- | --- |
| 1. It isn't clear if this is a project focused on improving assessment of nerve injury patients and VCA recipients, or a project seeking to implement novel machine learning techniques. The manuscript as presented doesn't completely do either. | Thanks for the comments. This project is about better characterizing gait in nerve injury models. This study aims at doing a more comprehensive multivariate characterization of gait than has been done in the literature to date. It is then about demonstrating that the multivariate relationships uncovered in the process have validity to them suggested by the fact that using the uncovered relationships resulted in superior performance when used to train a classifier (compared to classifiers that just used relationships uncovered via univariate analysis). Furthermore, the trained classifier was able to discriminate between different pathological gait states with a high degree of performance. A comprehensive revision of the manuscript to better reflect this throughout was completed.  Excerpts from the revised manuscript that aim to better reflect this are included below for convenience (these are taken from throughout the Abstract, Introduction, Results, and Discussion):  *“Animal models of nerve injury are important for studying nerve injury and repair, particularly for interventions that cannot be studied in humans. However, the vast majority of gait analysis in animals has been limited to univariate analysis even though gait data is highly multi-dimensional. As a result, little is known about how various spatiotemporal components of the gait relate to each other in the context of peripheral nerve injury and trauma. We hypothesize that a multivariate characterization of gait will reveal relationships between spatiotemporal components of gait with biological relevance to peripheral nerve injury and trauma. We further hypothesize that legitimate relationships between said components will allow for more accurate classification between distinct gait phenotypes than if attempted with univariate analysis alone* (lines 199-213) *… This is the first performance evaluation of a multivariate approach to gait analysis in animals. It is also the first study to use multivariate statistics to characterize and distinguish between different gradations of gait deficit in animals. This study contributes a comprehensive, multivariate characterization pipeline for application in the study of any pathologies in which gait is a quantitative translational outcome metric* (lines 233-292) *…* *There are widely adopted methods for evaluating success when studying nerve injury and repair. These include methods for assessing cellular appearance (histopathologic examination) (4), nerve conduction (nerve conduction velocity) (5), nerve-muscle connection (electromyography) (6), sensory function (Hargreaves) (7), and motor function (Rotarod, DigitGait, CatWalk, swimming, climbing) (8, 9)* (lines 311-330) *… limited, univariate gait studies span a variety of rodent models of gait deficit including those of metabolic (diabetic neuropathy) (22), degenerative (ALS) (23), traumatic (nerve transection / limb transplantation) (24), congenital (25), and other etiologies. While these diseases may result in dysfunctional gait, it is unlikely that the spatiotemporal details of their dysfunction are the same. For locomotion, indices such as the Sciatic Function Index (SFI) have been developed via multivariate linear regression models to compare healthy and dysfunctional gait (26). However, the SFI is a dimensionless number and does not reveal additional insight into exact relationships between specific spatial and temporal measures (27). In short, what does it really mean if the SFI index is statistically different between two gait states? How do we describe more than just a “difference” between two states?* (lines 348-401) *… The goal of this study was to use multivariate statistics to learn about the features, relationships, and factors that are most descriptive of gait in different pathological states. In doing so, we explored the hypothesis that a multivariate characterization of gait will reveal novel relationships between spatiotemporal components specifically in the context of peripheral nerve injury and trauma* (lines 1151-1155) *… We hypothesized that these relationships were deterministic of specific phenotypes, which contrasts with features that are not related to describing specific phenotypes and may conversely model noise* (lines 1167-1169) *… In* ***Fig 3*** *the wrapper-based, multivariate feature selection resulted in the usage of 10 to 15 select features in the model and thereby decreased the MCE of the model by more than 20%. This result supports the hypothesis that accounting for relationships between components of the gait would allow for more accurate classification between distinct gait phenotypes than univariate studies alone. This accounting for multivariate relationships is largely absent from the field of animal gait analysis* (lines 1179-1819) *… The level of exploratory statistical description in the prior few paragraphs is largely absent from studies across the field, including some of the latest, that employ gait analysis (18, 25, 37). A recent study contributes evidence toward using gait as a quantitative translational outcome metric for therapeutic development in Angelman syndrome and other genetic neurodevelopmental syndromes (25). This study contributes a comprehensive, multivariate characterization pipeline for application in the study of any pathologies in which gait is a quantitative translational outcome metric* (lines 1555-1561) *… the specific measures of the paw are consistent with biological differences in the respective pathologies. For example, limb transplantation injury was better characterized by the relationship of the paw to the midline, which may be due to loss of mechanical integrity and thereby positioning of the limb in relation to the area surrounding the transplant itself. In contrast, the nerve transection injury model was better characterized by fine motor movements of the paw (i.e. MIN dA/dT). This suggests that finer measures are more discernible when there is only nerve injury as compared to nerve + muscle + bone injury (****Table 2****). The results from factor analysis were consistent with the results from sequential feature selection and helped validate that the two gradations of peripheral nerve injury shared numerous important features. The results also displayed key differences related to fine vs. gross motor movements in each model respectively. Factor analysis allowed for a deeper understanding of the exact relationships among features in contrast to a simple rank list of features as provided by many feature selection techniques. This level of statistical description is currently lacking from gait studies in animal models. The many insights revealed support the hypothesis that multivariate gait analysis provides a more comprehensive statistical description of individual gait states than univariate analysis …* (lines 1590-1616)*”* |
| 2. There are multiple grammar/usage errors. For example, in the abstract on line 74, "Not all interventions can be experimented in humans". I assume the authors mean not all interventions can be studied in humans. The following sentences which state "methods for evaluating rodent gait lack the multivariate description applied to humans limiting their effectiveness. The authors then go on to say that multivariate evaluation of murine gait models is a novel clinical problem. This statement is not clear, and examples like this are common in the submitted manuscript. | Thank you for your patience in reading through the grammage / usage errors. A comprehensive read of the manuscript to identify and correct grammatical / usage errors was completed. |
| 3. There is no clear hypothesis that will be tested. This should be added to the Objective of the abstract. Was the hypothesis that multi-variate analysis is better than univariate analysis? Why was a hind limb transplant model used? Did the authors hypothesize that multivariate analysis would detect deficits better than univariate analysis in transplants than in nerve resection models? Was that the case? The only proof I saw was that more factors were identified by a more complex analysis. Does that empirically mean it is better? | The hypotheses to be tested have been clarified and added to the objective and throughout the manuscript. Specifically they are:   - **Hypothesis 1:** A multivariate characterization of gait will reveal relationships between spatiotemporal components of gait that are biologically consistent with each pathological state of peripheral nerve injury and trauma studied in the manuscript.   **Evidence:**   - **Table 2** shows the latent factors and the features they are composed of for pathological gait due to limb transplantation. **Table 2** also shows the latent factors and the features they are composed of for pathological gait due to nerve transection injury. The bolded latent factors in each sub-table (Factor 4 and Factor 6 respectively) are composed of different features among the two groups. The other 5 latent factors are composed of the same (or highly similar) features. The distinction is that pathological gait due to limb transplantation is distinguished by the relationship between the absolute paw angle and midline distance. Pathological gait due to nerve transection injury is distinguished by the relationship between paw area at peak stance in sq. cm, paw area variability at peak stance in sq. cm, and MIN dA/dT. The high degree of overlap of feature groupings among the two pathologies suggests that much of the statistical description of gait in these two sources of traumatic nerve injury is the same with a significant point of difference being factors 4 and 6 respectively (i.e. the two bolded columns) (**Table 2**). - This suggests that animals with limb transplantation have more variation in the anatomy of their transplanted paw in relation to the midline of their bodies. In contrast, animals that received nerve transection injury and reattachment do not demonstrate this same variation. This is consistent with the underlying biology: animals with a total limb transplantation likely have an alteration in the anatomical configuration of their paw related to their midline while animals receiving only nerve transection injury might not. Additionally, the data suggests that animals with exclusively nerve transection injury have a discernible variation in how the contact of their paw relates to their ability to make fine motor movements (i.e. jerks or dA/dT) while animals receiving a total limb transplant are likely to have this masked by gross motor defects related to the extensive musculoskeletal injury that accompanies the nerve injury from the transplant. - **Hypothesis 2:** Pathological gait due to different forms of traumatic injury could be characterized by certain latent factors that are determined by and composed of the selected features.   **Evidence**: see above paragraphs   - **Hypothesis 3:** There is a critical subset of features that more accurately describes healthy versus pathological gait. And that the subset identified via multivariate characterization more generalizably describes gait when compared to the subset identified via univariate characterization.   **Evidence**:   - **Fig 3**, illustrates how a simple, univariate feature addition approach, using 30 features minimized MCE. Ultimately, the smallest MCE achieved with this method was 0.17 indicating a potential model accuracy of 83% in discriminating healthy gait from pathological gait. However, this is a model trained on one holdout set with likely overfitting from using 30 of the 32 available features. It is likely that this is modeling noise. - Multivariate, sequential feature selection was used to sequentially add features in various relationships to the model and monitor exactly which feature relationships lead to decreases in MCE. Iteratively doing so until MCE was minimized.  When classifying between healthy gait and pathological gait, our results show that 10 to 16 features were optimal to minimize MCE (smallest MCE achieved = 0.10) (**Fig 3**). . This is a 5-7% improvement in MCE achieved using ~20 less features than the univariate approach above. Note how MCE increases beyond the critical numbers of features indicating overfitting and supporting the hypotheses that: (a) there is a critical subset of features that more accurately describes healthy versus pathological gait and (b) the subset identified via multivariate characterization more generalizably describes gait when compared to the subset identified via univariate characterization. - **Hypothesis 4:** Using the identified relationships between components will allow for more accurate classification between distinct gait phenotypes than if attempted with univariate analysis alone.   - **Evidence**:     - See above paragraphs - **Hypothesis 5:** Characteristic feature relationships of distinct gait phenotypes could be used to train a classifier capable of distinguishing between said phenotypes.   - **Evidence:** Using the features identified via multivariate feature selection and confirmed by factor analysis we assessed four different model architectures (**Table 3**). Accuracy in distinguishing between healthy gait and pathological gait due to any form of peripheral nerve injury (**Table 3a**) ranged from .73-.91, precision from .76-93, recall from .75-.91, and F-Score from .75-.92. The ensemble model had the highest performing overall metrics in distinguishing between healthy gait and pathological gait due to any form of peripheral nerve injury**.**   - Accuracy in distinguishing between types of peripheral nerve injury (i.e. hind limb transplant vs. total nerve transection) ranged from .64-.88, precision from .71-.93, recall from .69-.88, and F-score from .69-.90. Again the Ensemble model was the overall highest performing architectures in distinguishing between different types of peripheral nerve injury.   Scores outputted by the model on the holdout set were graphed as a box and whisker plot. A strong statistical difference in score distribution between healthy versus injured animals as well as between pathological states was observed (p<.0001) (**Fig 5**). |
| 4. The authors need a better description in the methods section of what machine learning, and training of classifier models is and what it means in relation to normal animals, nerve transection and hind limb transplant models. | A description of machine learning and training classifier models as it relates to each group has been added to the methods section. Excerpt included below for convenience:  *“Definitions Within Study Context*  *Machine learning: computational statistical algorithms that learn from data labeled as coming from healthy, nerve transected, or limb transplanted mice and using those learnings to understand the features and relationships that encode patterns specific to those respective groups to enable the training of models that can be used for various purposes like classification.*  *Training of classifier models: trained a computational statistical model via machine learning methods using data labeled as coming from healthy, nerve transected, or limb transplanted mice. The classifier models are encoded by features and relationships identified by the machine learning methods. Once trained, new, unlabeled data from animals of uncertain gait quality can be input into the model. The model will then return numerical values representing the likelihood that the input data is characteristic of a healthy, nerve-transected, or limb-transplanted animal and its gait. The output values can be used to classify animals with known identities to quantify the quality of the statistical characterization conducted on each gait phenotype.”* (lines 721-734) |
| 5. Based on clinical experience to date, it is unlikely that clinical lower limb transplantation will become common. Results with lower limb prosthetics are just too good. The authors might want to relate how the proposed work might benefit upper limb transplantation. | Response: Agreed. The utility of the hind limb transplantation model in this paper is four-fold:   1. As part of an increasing gradient of damage on which to apply and validate the ability of multivariate characterization to identify biologically relevant features and relationships. 2. The demonstration of the value of multivariate characterization as a novel and proposed new standard for gait analysis in an intervention + model that is difficult albeit impossible to study in humans. 3. The value of establishing such a model and characterization pipeline to facilitate research that may also benefit upper extremity transplantation. 4. To maximize the degree of damage and thereby spatiotemporal changes to the limb in the interest of evaluating this new method of gait characterization at its limits   All three of these benefits have been clarified and added to the manuscript, particularly to the method section as quoted here:  *“Additionally, while clinical experience to date suggests it is unlikely that clinical lower limb transplantation will become common, the hind-limb transplantation model was still used in this study for four main reasons: (1) as part of an increasing gradient of damage on which to study the utility of multivariate characterization to identifying biologically relevant features and relationships, (2) to investigate the value of multivariate characterization as a novel and proposed new standard for gait analysis in a therapeutic intervention and model that is difficult, albeit, impossible to study in humans, (3) to establish and characterize this model and statistical pipeline as one of potential benefit to research in functional nerve recovery for upper extremity transplantation, and (4) to maximize the degree of damage and thereby spatiotemporal changes to the limb in the interest of evaluating this new method of gait characterization at its limits.”* (lines 510-561) |
| 6. Pay attention to grammar, another example, line 138 - "peripheral nervous injury" - should be peripheral nerve injury | Thank you for catching this, it has been fixed and a comprehensive read of the manuscript to fix grammatical errors was completed. |
| 7. Be careful about rationale for using male mice in methods section. The statement that male mice were used, because in the author's experience "they appear to be more tolerant to hindlimb transplant surgery" is a difficult statement to defend | Agreed, this statement has been removed. |
| 8. Figure 2 - comparing normal function to nerve resection or a transplant and looking for differences is not difficult. The authors state that only 44% had a p value below the 0.001 threshold. Why is this not higher? State clearly what the authors expected and why it is important to show this data | Agreed, looking for differences via t-test and multiple hypothesis testing is the univariate analysis done across the field. This was done as a baseline so we may replicate what typical, less sophisticated methodologies (i.e. non-multivariate) analysis of gait is performed across the field. 44% does not represent 44% of all observations (i.e. animals). 44% means that 44% of the measured spatiotemporal parameters (i.e. features) across all observations were found to have a statistical difference. The importance of showing this data is to set a baseline of what univariate analysis would find, to use the significant features identified via univariate analysis to evaluate model classification accuracy, and then evaluate the hypothesis that, if, multivariate characterization identifies feature sets and feature relationships that better describe gait states, that it would lead to more accurate classification. This has been clarified in the text as shown in the quoted excerpt below:  *“In this study we implement a simple filter by applying univariate criteria separately on each feature with Bonferroni correction. This was done as a baseline to replicate typical (i.e. non-multivariate) analysis of gait performed across the field. This baseline is important for evaluating the hypothesis that multivariate characterization identifies feature sets and feature relationships that better describe gait states and lead to more accurate classification.”* (lines 642-646) |
| 9. The same lack of purpose is exhibited in table 1. Is the point that there are more features with multivariate analysis? The statement "Within each type of of analysis (univariate vs. multivariate), the two pathological states have high degree of overlap with key differences among both pathological states" is very confusing. What are the authors trying to say? | This has been clarified in the caption of table 1 and is included here for convenience:  *“Based on the univariate analysis, we see three types of features in both pathological states: those related to phase (absolute and relative measures), measures of the paw, and measures of symmetry. Features selected from forward selection (multivariate analysis) in both pathological states revealing features related to stride length and how it varies, measures of the paw, measures of symmetry, and measures of phase. Features are listed in rank order based on their contribution to characterizing each pathology as selected by the forward selection algorithm. Bolded features are ones that are shared between phenotypes. Notice that univariate and multivariate analysis have significant differences in the relevant features with certain areas of overlap. Within each type of analysis (univariate vs. multivariate), the two pathological states share numerous features. The features identified via univariate and multivariate characterization respectively will be used to build classifier models to quantify our ability to classify between gait states in the subsequent figures and tables.”* |
| 10. Please go through the rest of the manuscript and clearly state the reason for the analysis, and how it relates to the hypothesis that will be presented in the abstract. | Thank you for helping to make the rationale for doing each analysis clearer. Now, each analysis method has a clear reason stated with accompanying relation to the relevant hypothesis it explores as quoted in the following excerpts from the manuscript:  *“the vast majority of gait analysis in animals has been limited to univariate analysis even though gait data is highly multi-dimensional. As a result, little is known about how various spatiotemporal components of the gait relate to each other in the context of peripheral nerve injury and trauma. We hypothesize that a multivariate characterization of gait will reveal relationships between spatiotemporal components of gait with biological relevance to peripheral nerve injury and trauma. We further hypothesize that legitimate relationships between said components will allow for more accurate classification between distinct gait phenotypes than if attempted with univariate analysis alone* (lines 200-213) *… In human gait analysis, studies have investigated multivariate techniques to describe relationships between measurable features of gait (1). These relationships help statistically characterize gait phenotypes and are used to select features for measurement, classification, and prediction (1). Implementing a similar level of statistical rigor to animal gait studies represents a novel problem requiring further refinement and investigation* (lines 331-336) *… In rodents, the study of locomotion via treadmill gait (DigiGait™) or free ambulating (CatWalk™) systems provides high levels of spatial and temporal detail, measuring over 30+ individual spatial or temporal gait parameters (10-14). Analyses of data measured by these systems are largely limited to traditional, univariate, multiple-hypothesis testing or feature selection (15-18). Usually this involves comparing the means of individual output parameters for statistical significance and applying some correction for multiple-hypothesis testing (19, 20). Such univariate study enables conclusions that do not account for possible relationships between individual gait parameters. A machine learning model can also be trained using feature selection techniques that omit the examination of multivariate relationships between features (21). Thus, discoveries in rodent models, to date, have been primarily limited to a portion of the interplay that makes up the animal’s overall gait* (lines 337-347) *… These limited, univariate gait studies span a variety of rodent models of gait deficit including those of metabolic (diabetic neuropathy) (22), degenerative (ALS) (23), traumatic (nerve transection / limb transplantation) (24), congenital (25), and other etiologies* (lines 348-350) *… A comprehensive description of the rodent’s gait would require a methodology that can factor relationships among all 30+ measures and prioritize those that contribute to gait phenomena rather than noise. We hypothesize that out of all spatiotemporal measures of rodent gait, there exists a discrete subset sufficient to uniquely describe the gait of three distinct physiological states: (1) healthy animals, (2) those with nerve injury due to a complete nerve transection and re-attachment, and (3) those with nerve, muscle, and bone injury due to total limb transplantation. Furthermore, we hypothesize that multivariate characterization will reveal relationships that encode and describe the different gait phenotypes in a biologically consistent manner. Finally, we hypothesize that the revealed relationships will enable the training of a classifier model that can discriminate between gait states with higher accuracy than models trained with features identified via univariate analysis* (lines 402-412) *… 43 male B6 mice, 8 to 10 weeks old (20g), were obtained from The Jackson Laboratory (Bar Harbor, ME, USA) and were group-housed. Male mice were used due to their larger size, thereby having easier vascular access. There were three groups: 17 animals in the negative control group received no treatment. 12 animals in the experimental group received only nerve damage (neurorrhaphy) i.e. a complete nerve transection of the femoral and sciatic nerves with re-coaptation. 14 animals in the second experimental group received a total hind-limb transplant* (lines 487-493) *… The motivation for assessing these three groups was to establish a gradient of increasing neuromuscular damage. In doing so, the goal was to characterize how spatio-temporal gait parameters were altered accordingly. Thereby, investigating how musculoskeletal damage, in addition to nerve injury, alters the statistical characterization compared to just the contribution of nerve injury alone. In other words, groups were chosen to represent increasing degrees of damage to the neuromusculoskeletal sequence of healthy gait* (lines 495-500) *… Exploratory analysis to reduce and identify the primary dimensions responsible for describing rodent gait was conducted via feature selection and factor analysis techniques* (lines 628-630) *… In this study we implement a simple filter by applying univariate criteria separately on each feature with Bonferroni correction. This was done as a baseline to replicate typical (i.e. non-multivariate) analysis of gait performed across the field. This baseline is important for evaluating the hypothesis that multivariate characterization will identify feature sets and feature relationships that better describe gait states and will lead to more accurate classification.* *We also apply a multivariate, forward sequential feature selection in a wrapper fashion to find important features with the goal of minimizing misclassification error (MCE) of our learning algorithm. This was done to explore the hypothesis that multivariate characterization will reveal relationships that encode and describe the different gait phenotypes in a biologically consistent manner unlike univariate analysis; and that the revealed relationships will enable the training of a classifier model that can discriminate between gait states with higher accuracy than models trained on features identified via univariate analysis. Embedded selection was then applied during model training to further confirm the ideal feature set* (lines 634-653) *… To learn more about the exact relationships among features, factor analysis was conducted. Understanding the exact relationships among features is important to exploring the hypotheses that said relationships encode and describe the different gait phenotypes in a biologically consistent manner; and that the revealed relationships will enable the training of a classifier model that can discriminate between gait states with higher accuracy than models trained with features identified via univariate analysis* (lines 658-663) *… Models trained via multivariate feature selection were compared for their performance with a model trained via univariate feature selection to explore the hypothesis that a classifier model trained on features identified via multivariate analysis can discriminate between gait states with higher accuracy than models trained with features identified via univariate analysis.* (lines 751-755)*”* |
| 11. In figure 5, Why do healthy animals have scores close to no function, (1 = fully functional, 0 = no function)? Figure 5 also highlights the fact that nerve function after only two weeks of healing is very limited. Better nerve function would be seen at one month. Did the authors want animals with incomplete nerve recovery, and if so, what was the rationale? | We apologize for the confusion. To clarify, the injury “score” is simply a statistical output to use in delineating whether an animal is likely to be part of group 0 (experimental = transplant or transection ) or group 1 (healthy control). Thresholding must then be done to identify the most statistically justifiable line. In this case, statistics were run between the distributions of outputs to evaluate if the distributions were in fact statistically different from each other, which in all three comparisons, they were (Figure 5). The figure itself is a box and whisker plot, so the boxes indicate the interquartile range and are more indicative of a wider variance among outputs rather than the presence / concentration of outputs. This is likely due to variation seen in the data collection period, which would be consistent with the fact that animals are freely allowed to move however is natural to them on the treadmill and that the data collection is done from video cameras. On one hand, collected data will naturally be less precise, on the other hand, this data collection lends itself to greater flexibility as it has less stringent experimental constraints. Yes, there is value to having incomplete nerve recovery to allow the statistical characterization to evaluate, with greater resolution, differences between animals with nerve injury versus animals with nerve + muscle + bone injury. Moreover, because timing of data collection was consistent across groups, and the focus is to compare how the multivariate characterization described differences between the groups, we felt further supported in the time point that was picked. We have proceeded to adjust the use of the word “score” accordingly in the text. |

**Reviewer #2**

| **Comment from Editor / Reviewer** | **Responses and Quoted Excerpts from Manuscript** |
| --- | --- |
| 1. The introduction of the article is quite long and needs to be shortened | The introduction has been shortened. Moreover, we included details felt necessary to set the stage for this work that has little comparable work in the literature to draw from. Though, there is significant precursor work in the literature to set the stage with, thus, leading to a longer introduction. We felt comfortable in doing so as PLOS One specifies not having strict word requirements. |
| 2. The hypothesis of the article should be stated more clearly in the introduction | Done, as shown in this quoted excerpt:  *“We hypothesize that out of all spatiotemporal measures of rodent gait, there exists a discrete subset sufficient to uniquely describe the gait of three distinct physiological states: (1) healthy animals, (2) those with nerve injury due to a complete nerve transection and re-attachment, and (3) those with nerve, muscle, and bone injury due to total limb transplantation. Furthermore, we hypothesize that multivariate characterization will reveal relationships that encode and describe the different gait phenotypes in a biologically consistent manner unlike univariate analysis. Finally, we hypothesize that the revealed relationships will enable the training of a classifier model that can discriminate between gait states with higher accuracy than models trained with features identified via univariate analysis.”* (lines 404-412) |
| 3. There seems to be an inconsistency between the title of the study and the hypothesis of the study. While the study title states "Multivariate description and scoring of neuromotor changes in a mouse model of peripheral nerve injury", the study seems to test the validity and reliability of a video analysis program called DigiGait. Therefore, it seems necessary to change either the title or the hypothesis of the article or the content of the study. | Good point - we’ve adjusted the title for greater precision to “Multivariate description of gait changes in a mouse model of peripheral nerve injury and trauma” |
| 4. It is not understood why isotransplantation was preferred over autotransplantation in the study. However, autotransplantation is the first choice in limb amputations in the clinic | We concur. In the clinic, autotransplantation can be done and is the first choice when the amputated limb is in a reasonable shape with well-preserved muscular integrity, vasculatures and minimal nerve damage. However, it is difficult to do in small animals like in rats and even more so in mice, so, we choose isotransplantation as the closest proxy for autotransplantation in which to study transplant injury (free of rejection response). There are needs for allotransplantation (MHC-mismatched), which is when the amputated limb is in bad condition and not possible to reconnect. |
| 5. In addition, histopathologic examination methods of neural recovery were not included in the study. Therefore, the study does not include detailed recovery data including the histopathological and biochemical examinations of the subjects who underwent transection and limb transplantation. | The point is well taken. We focused on the gait changes and analysis in this paper, while extensive histopathological and biochemical examinations are ongoing. In the revised manuscript, we have specified in the title and elsewhere to focus on “gait changes” rather than broader “neuromotor changes”. |
| 6. Many other neuromotor analysis methods (such as swimming, sciatic function test, and climbing) were also not included in the study. In addition, target organ (such as gastrocnemius muscle) involvement levels were not included in the study. Therefore, detailed analysis of neuromotor recovery was not performed | We appreciate the precision. Same as above, we have adjusted the paper to focus on gait changes and analysis. For example, we have specified in the title and elsewhere that the paper that the paper is focused on gait changes specifically and not broader neuromotor changes. The new title states “Multivariate description of gait changes in in a mouse model of peripheral nerve injury and trauma”. |
| 7. In addition, there are already studies similar to this study in the literature as follows: Ganguly A, McEwen C, Troy EL, Colburn RW, Caggiano AO, Schallert TJ, Parry TJ. Recovery of sensorimotor function following sciatic nerve injury across multiple rat strains. J Neurosci Methods. 2017 Jan 1;275:25-32. doi: [10.1016](tel:101016)/j.jneumeth[.2016.10](tel:201610).018. Epub 2016 Oct 29. PMID: [27984099](tel:27984099) | The identified study explores the relationship between animal strain and sensorimotor function over time. The SFI and proprioceptive hind limb placement (PHLP) are merely two measures that the DigiGait system outputs. While the SFI is inherently multivariate to a limited degree, it is not as comprehensive as the multivariate characterization we have conducted in this study where we account for and evaluate all 30+ spatiotemporal measures collected by the DigiGait system. We have included this study as a citation within and also have included the value of our study in comparison to the SFI as shown in this quoted excerpt below:  *“For locomotion, indices such as the Sciatic Function Index (SFI) have been developed via multivariate linear regression models to compare healthy and dysfunctional gait (26). However, the SFI is a dimensionless number and does not reveal additional insight into exact relationships between specific spatial and temporal measures (27). In short, what does it really mean if the SFI index is statistically different between two gait states? How do we describe more than just a “difference” between two states?”* (lines 352-401) |

**Reviewer #3**

| **Comment from Editor / Reviewer** | **Responses and Quoted Excerpts from Manuscript** |
| --- | --- |
| 1. The first sentence in the abstract (lines 72-73) is not clear. “Quantitative scoring enabling comparison of neuromotor recovery in peripheral nerve injury models is critical to understanding and gauging the extent of injury and repair.” Just add commas to the sentence to be more readable and to separate the introductory phrase from the main clause. “Quantitative scoring, enabling comparison of neuromotor recovery in peripheral nerve injury models, is critical to understanding and gauging the extent of injury and repair.” | Agreed, thank you for the suggestions, a comprehensive read of the manuscript with adjustments for clarity and readability was completed. This includes the separation of longer sentences or the addition of commas as needed. |
| 2. Lines 97-98 are not enough for the conclusion. You may need to add briefly the contributions and advantages for your proposed solution. | Thanks for the suggestion. We have added additional detail to the conclusion portion of the abstract as suggested and quoted here for convenience:  *“This is the first performance evaluation of a multivariate approach to gait analysis and the first demonstration of superior performance as compared to univariate gait analysis in animals. It is also the first study to use multivariate statistics to characterize and distinguish among different gradations of gait deficit in animals. This study contributes a comprehensive, multivariate characterization pipeline for application in the study of any pathologies in which gait is a quantitative translational outcome metric.”* (lines 233-292) |
| 3. The introduction was written in a clear way, and the authors presented several works in the literature. It is better to add at least a recent reference from the last year | Thank you – we have added this recent gait analysis from 2023: Lu Y, Lin Z, Li M, Zhuang Y, Nie B, Lei J, et al. Three-phase Enriched Environment Improves Post-stroke Gait Dysfunction via Facilitating Neuronal Plasticity in the Bilateral Sensorimotor Cortex: A Multimodal MRI/PET Analysis in Rats. Neurosci Bull. 2023. |
| 4. You should add references to the two approaches used in Lines [227-229](tel:227229) | Done with the following reference: Remeseiro B, Bolon-Canedo V. A review of feature selection methods in medical applications. Comput Biol Med. 2019;112:103375. |
| 5. In line 254, you mentioned that the same animal was not included in both the training and test sets within a respective fold. How did you ensure that using randomly 10-fold cross validation? | This is in reference to the fact that DigiGait data is output as data pertaining to each limb of a single animal contained within its own row. So, to clarify, this is in reference to ensuring that grouping into training and test sets was done at the organism level and not the individual record level to prevent the model training on patterns specific to individual organisms. |
| 6. Authors should describe the dataset in more details. How many samples in the dataset? What is the number of samples of each class (healthy, surgery)? What is the number of mice that participated in the experiment? These are not mentioned in the methodology section | Agreed - this has been included in the methodology section as quoted here: *“There were three groups: 17 animals in the negative control group received no treatment. 12 animals in the experimental group received only nerve damage (neurorrhaphy) i.e. a complete nerve transection of the femoral and sciatic nerves with re-coaptation. 14 animals in the second experimental group received a total hind-limb transplant. Animals receiving a procedure received only one procedure on their left hind limb.”* (lines 490-494) |
| 7. Authors should describe in more detail the data that is processed in the classifiers. How many input characteristics are there? | Response: 16 input characteristics were included in the classifiers, this has now been specified within the methods section as quoted here: *“Based on the feature selection results 16 features were chosen to be included in model training”* (lines 744-745) |
| 8. What are the hyperparameters that were used in each classifier (e.g. C and gamma in SVM, number of trees in Random Forests…etc.)? | Hyperparameter tuning with “auto” (i.e. default) settings was performed. In more detail, for SVM, the kernel function was linear and thus there is no gamma value. For the C hyperparameter, a value of 1 was used. |
| 9. Did you perform hyperparameter tuning to select the best parameters for each model? This may improve the performance. | Yes, hyperparameter optimization was performed for each model. |
| 10. It is better to add the confusion matrix of the results and appropriate evaluation metrics. | Thanks for the suggestion, we have calculated confusion matrices and included as supplemental information. |
| 11. Maybe it is worth testing other ensemble-based classifiers (e.g. XgBoost or stacking classifiers) to improve the performance. | This is a good idea. We tested an ensemble-based classifier and added its performance metrics to the table. Performance was indeed improved as shown below |

| **A. Performance in Distinguishing Peripheral Gait Deficit from Healthy Gait** | **Accuracy** | **Precision** | **Recall** | **F-Score** |
| --- | --- | --- | --- | --- |
| **Random Forest** | 0.7294 | 0.7560 | 0.7521 | 0.7492 |
| **Discriminant Analysis** | 0.7477 | 0.8022 | 0.7634 | 0.7742 |
| **Support Vector Machine** | 0.7744 | 0.8108 | 0.7915 | 0.7948 |
| **Regression** | 0.7868 | 0.8570 | 0.7826 | 0.8130 |
| **Ensemble** | **0.9099** | **0.9283** | **0.9086** | **0.9165** |
| **B. Performance in Distinguishing Between Two Phenotypes of Peripheral Gait Deficit: Limb Transplant from only Nerve Transection** | Accuracy | Precision | Recall | F-Score |
| **Random Forest** | 0.6435 | 0.7072 | 0.6852 | 0.6878 |
| **Discriminant Analysis** | 0.7165 | 0.7827 | 0.7188 | 0.7388 |
| **Support Vector Machine** | 0.6987 | 0.7806 | 0.7341 | 0.7457 |
| **Logistic Regression** | 0.7237 | 0.7882 | 0.7270 | 0.7456 |
| **Ensemble** | **0.8780** | **0.9263** | **0.8781** | **0.8984** |
