# Supplementary material for: Multivariate description of gait changes in a mouse model of peripheral nerve injury and trauma
Source: PLoS One. 2025 Jan 7;20(1):e0312415. doi: 10.1371/journal.pone.0312415 (PMC11706367; doi:10.1371/journal.pone.0312415)
Supplement: Attachment — Submitted filename: Rebuttal Letter.docx [file pone.0312415.s003.docx]

PONE-D-23-36233R1

Multivariate description of gait changes in a mouse model of peripheral nerve injury and trauma

PLOS ONE

Dear Editor,

Thank you for the reviewers’ comments and suggestions. Our responses are the following:

Review Comments to the Author

Reviewer #4: Well written study addressing a notable gap in current PNI research and incorporation of machine learning to further minimize observational bias. The authors propose that a multivariate analysis of gait could uncover significant connections between spatiotemporal aspects of gait that are biologically pertinent to PNI. They further propose that these relationships will enable a more precise identification of distinct gait patterns compared to using only univariate analysis.

The authors have addressed all previous comments and concerns raised by the reviewers. Yet, concern still remains in generalizing the authors hypothesis to current gait research protocols within the scientific community. This issue may benefit from further discussion.

**Response:**

We are grateful for the opportunity to further discuss the generalizability of the hypothesis to current gait research protocols within the scientific community. We have added a few paragraphs to the Discussion section to address this as excerpted below. The additions also include references to a number of studies that help build support for the potential generalizability of the approach:

(page 24) “The level of exploratory statistical description in the prior few paragraphs is largely absent from rodent studies across the field, including some of the latest that employ gait analysis [18, 25, 37]. Thus, we hypothesize that multivariate statistics has generalizable potential in reducing observational bias to current pre-clinical gait research protocols in the scientific community.

Towards this hypothesis, a recent study contributes evidence toward using gait as a quantitative translational outcome metric for therapeutic development in Angelman syndrome and other genetic neurodevelopmental syndromes [25]. This study contributes a comprehensive, multivariate characterization pipeline for application in the study of any pathologies in which gait is a quantitative translational outcome metric. Another study was comparable in its level of statistical description [31], coincidentally doing so in a genetic neurodevelopmental model of cerebellar ataxia [31]. Lambert et al. supported the value of multivariate characterization of gait by conducting feature extraction on highly dimensional gait data from a central nerve injury model of cerebellar ataxia. The authors hypothesized certain indirect, common factors that characterize gait and underlie the more directly measurable features. These common factors included thrust, rhythmicity, and contact area and were directly useful in discriminating between animals with a central lesion vs. those without [31]. In this study, we observed potential patterns in the selected features and hypothesized that pathological gait due to traumatic nerve injury could also be characterized by certain latent factors that are determined by and composed of the selected features.

(page 26) “This study offers a methodology that may be applied to existing DigiGait datasets to conduct similar exploratory multivariate analysis to reduce observational bias from traditional univariate methods. Evaluating the generalizability of this methodology on other etiologies of gait deficit is the subject of a subsequent study… As a new methodology within rodent gait research protocols, one limitation of this study is its limited scope. On one hand, the multivariate statistics demonstrated in this study offer a convenient way for researchers to conceptualize meaningful groupings of features. On the other hand, clear biological tie-ins and strong statistical significance in classifier performance when applied to gait research protocols across the scientific community stands to be evaluated. We have identified datasets of DigiGait data available at our Behavioral Phenotyping Core from investigators studying central causes of gait disorder (e.g. stroke, transient ischemic attack) for us to apply the pipeline to and are pursuing said inquiry…”
